# Congenital coronary artery anomalies in sports medicine. Why to know them

**DOI:** 10.1002/clc.24084

**Published:** 2023-07-11

**Authors:** Paolo Zeppilli, Massimiliano Bianco, Salvatore F. Gervasi, Michela Cammarano, Riccardo Monti, Fabrizio Sollazzo, Gloria Modica, Lorenzo Morra, Francesco M. Nifosì, Vincenzo Palmieri

**Affiliations:** ^1^ Sports Medicine Unit, Fondazione Policlinico Universitario A. Gemelli IRCCS Catholic University Rome Italy

**Keywords:** athlete, preparticipation screening, return to play, sport, sport related sudden death, sudden death

## Abstract

The anomalous origin of a coronary artery (AOCA) is a challenging topic, due to its rarity, the complexity of the pathophysiological aspects, the clinical presentation (often silent), the difficulty of diagnosis, and the potential risk of causing acute cardiovascular events up to sudden cardiac death, particularly when triggered by heavy physical exercise or sport practice. Increasing interest in sport medical literature is being given to this topic. This paper reviews current knowledge of AOCAs in the specific context of the athletic setting addressing epidemiological and pathophysiological aspects, diagnostic work‐up, sports participation, individual risk assessment, therapeutic options, and return to play decision after surgery.

## Graphical abstract

Diagnostic and therapeutic workup of high‐risk anomalous origins of coronary arteries (AOCAs) in athletes. AOCA, anomalous origins of coronary artery; CPET, cardiopulmonary exercise testing; CTA, coronary computed tomography angiography; ECG, electrocardiogram; FFR, fractional flow reserve; iFR, instantaneous wave‐free ratio; MRI, magnetic resonance imaging; NPI, nuclear perfusion imaging; TTE, transthoracic echocardiogram. The images used were downloaded, unchanged, from the link: https://mindthegraph.com/app

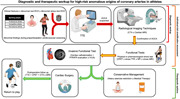

## 1 INTRODUCTION

The anomalous origin of a coronary artery (AOCA) is a challenging topic, due to its rarity, the complexity of the pathophysiological aspects, the clinical presentation (often completely silent), the difficulty of diagnosis and, at the same time, the potential risk of causing acute cardiovascular events up to sudden cardiac death (SCD).^1^, ^2^, ^3^

If we move the topic to the athletic setting, it becomes even more challenging. Nowadays, physical activity and sport practice is recommended at all ages, for healthy people and for a wide range of pathological conditions, so that an increasing portion of the population practices recreational sport. In addition, a top‐level athlete is a new hero of the modern era and when he or she suffers a sport related SCD, the individual and family drama becomes a social tragedy. Unfortunately, clinical cardiologists are often unaware of this entity, yet. Already more than 15 years ago, Paolo Angelini pointed out the need to increase the knowledge of this condition among clinical cardiologists, often unaware on the entity, the pathophysiology, the diagnostic workup, and risk assessment of the AOCA in sporting people.^2^

Aim of this paper is to review current knowledge of AOCAs in the specific context of the athletic setting. We focus mainly on the AOCAs arising from the “inappropriate,” opposite, coronary sinus. Moreover, as the course of the anomalous vessel impacts on the risk of SCD, we will focus only on AOCA from the opposite sinus of Valsalva with *an interarterial course/intramural course*, being this feature considered as more malignant.^1^, ^2^, ^4^, ^5^, ^6^, ^7^ Sports participation, individual risk assessment, therapeutic option, and return to play will also be addressed.

Before continuing in this paper, we need to be clear on the terminology to be used in the sporting context to avoid some confusion. In the following, we will define *competitive athletes* as individuals of school age and above (≥12 years of age) who regularly practice physical activity and participate in official competitions organized by a recognized Sports Federation or Association. Competitive and even more *professional* athletes place a high value on athletic excellence, and they typically train minimum 8−10 hours weekly with high exercise intensities. In contrast, we define *recreational athletes*, individuals who engage in recreational or leisure‐time sports activities on a regular or intermittent basis. Usually, they train much less and do not have the pursuit of excellence as their main purpose.^8^

## 2 EPIDEMIOLOGY

AOCAs are a rare, heterogeneous group of malformations, isolated or associated with other congenital cardiac defects. The prevalence of AOCA from the aorta in the general population is estimated to be 0.06%−0.9% for anomalous origin of right coronary artery (AORCA), 0.02%−0.1% for anomalous origin of left coronary artery (AOLCA), and 0.02%−0.6% for anomalous origin of circumflex artery,^9^, ^10^, ^11^ even if in a prospective study conducted with precise criteria and a rigorous classification, Angelini et al. reported a prevalence of 5.6%.^12^ These apparent discrepancies may be explained by the different methods used and the fact that from the anatomical point of view defining what is “normal” or “abnormal” in the coronary tree may be challenging. In addition, not all kinds of AOCAs show the same SCD risk, even during sport practice.

Focusing on high‐risk cardiac condition, Angelini et al. showed a 0.45% prevalence of interarterial AOCA in a large group of schoolchildren studied prospectively by means of rest electrocardiogram (ECG) and cardiac magnetic resonance imaging (MRI), with the more frequent anomaly (0.33%) being an interarterial AORCA from the opposite sinus.^13^ On the other hand, the interarterial AOLCA from the opposite sinus is more prevalent in autoptic series,^1^ confirming that the AOLCA is definitely more malignant.^1^, ^14^, ^15^

## 3 PATHOPHYSIOLOGY

Nowadays is widely demonstrated and accepted that heavy exercise can trigger SCD in AOCA's bearers. Corrado et al., in a milestone of the sports medical literature, showed that the relative risk for a SCD is actually 79‐fold higher in people with malignant AOCA during strenuous exercise than at rest.^16^ Initially, some authors^1^ attributed relevance to the hypothesis that the anomalous artery raising from the opposite sinus was compressed between the aorta and pulmonary trunk (“sandwich effect”) during exercises with increasing cardiac output and blood pressure. At the same time, other mechanisms were proposed to explain “ischemia” as an acute angle take‐off of the anomalous artery with functional closure of a slit‐like orifice, sometime with an ostial ridge and additional factors considered were the length, caliber, and position of the anomalous vessel in respect to the inter‐commissural pillar (above, under, or crossing it). More recently, the greatest importance has been attributed to *the intramural course* of the anomalous artery, especially its first tract which, being inside the aortic wall, would be compressed (obliterated) by the expansion of the aortic itself during systole.^4^, ^5^, ^17^ All these features may explain why patients with apparently similar anatomy have different clinical profiles and risk^1^, ^2^, ^6^, ^7^ and the approach must necessarily be individualized.

Based on the now extensive literature, the AOLCA with interarterial/intramural course seems to bring the higher risk of adverse events.^1^, ^10^, ^18^, ^19^, ^20^, ^21^ Other anomalies, such as a coronary artery arising from the opposite sinus but traveling anterior to the pulmonary artery (pre‐pulmonic) or posterior to the aorta (posterior/retroaortic), are generally considered benign.^22^ The same is deemed valid for the anomalous origin of left common trunk or the left anterior descending coronary artery alone from the inappropriate sinus running through the conal septum (intraseptal, intraconal, or intramyocardial),^7^, ^23^ although not all authors agree on its “absolute” benignity, as inducible myocardial ischemia and even cardiac arrest has been reported in single cases.^24^, ^25^ In addition, Doan et al. reported inducible myocardial ischemia during stress test in up to 50% of patients with intraseptal anomalous aortic origin of a coronary artery^26^ and even proposed a novel surgical approach to treat this condition.^27^

Finally, there is a substantial agreement on the benign prognosis, in young people and also in athletes, of the anomalous origin of the circumflex artery from the right sinus of Valsalva or from the right coronary artery with retro‐aortic course.^1^, ^2^, ^3^, ^28^, ^29^, ^30^, ^31^

## 4 DIAGNOSIS

**Figure 1:**
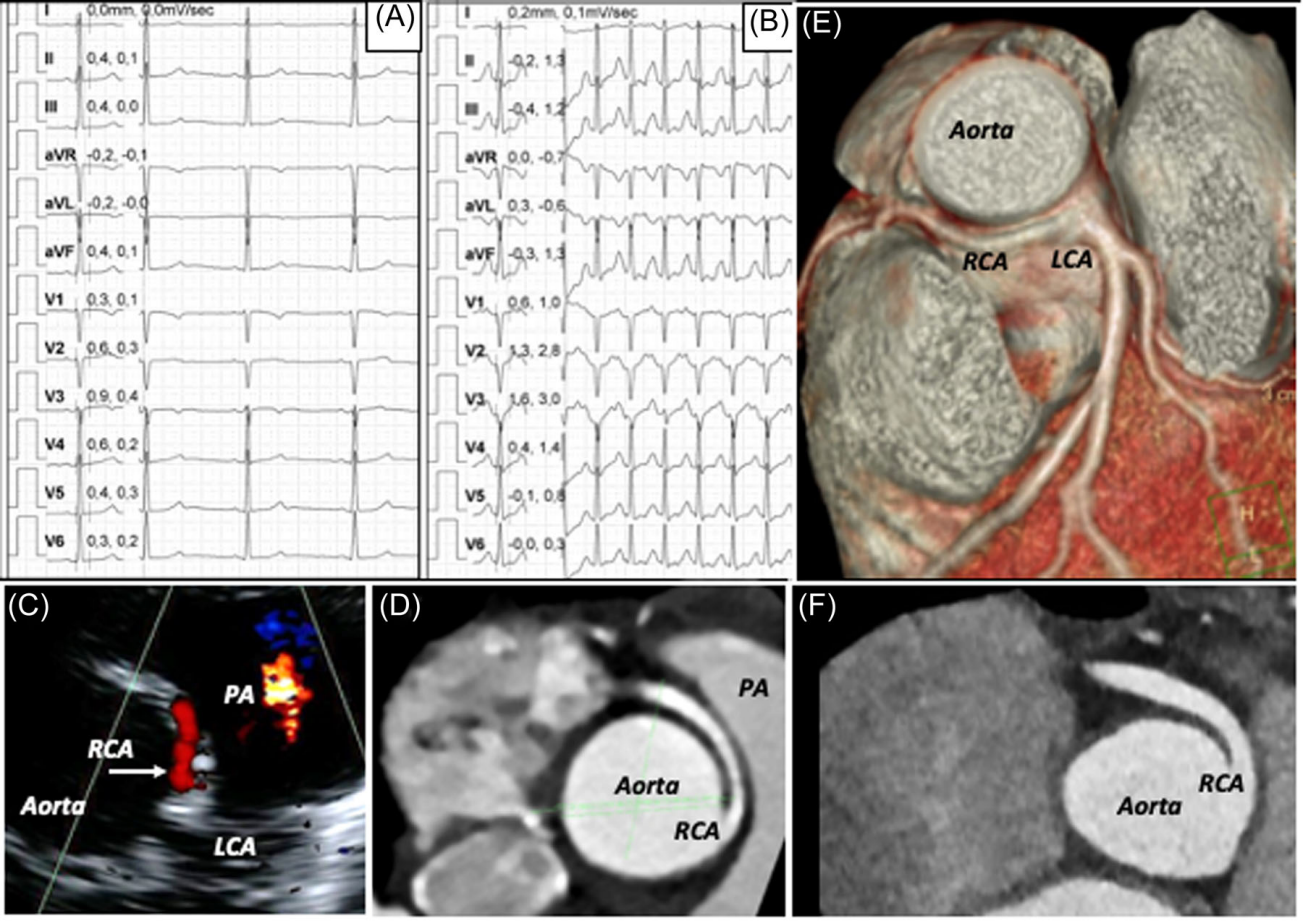
Twenty‐two year old female athlete, preparticipation screening for professional football. The rest electrocardiogram (A) was normal with an ST‐segment upsloping depression in the inferolateral leads at the stress test (B). The transthoracic echocardiogram (C) showed a diastolic color flow Doppler signal directed anteriorly between aorta and pulmonary artery, suggesting an anomalous origin of the right coronary artery with interarterial course. The suspicion was confirmed at the coronary computed tomography angiogram (D and E), which showed an hypoplastic proximal segment of the anomalous artery. Other malignant features were discovered during the risk stratification assessment and surgery was offered to this athlete, even if asymptomatic. The panel (F) shows the anatomical result after unroofing procedure. The athlete remained asymptomatic and after 3 months of reconditioning, with normal results at imaging and cardiopulmonary exercise testing, she resumed professional football (follow‐up 18 months). LAD, left anterior descending artery; PA, pulmonary artery; RCA, right coronary artery.

**Figure 2:**
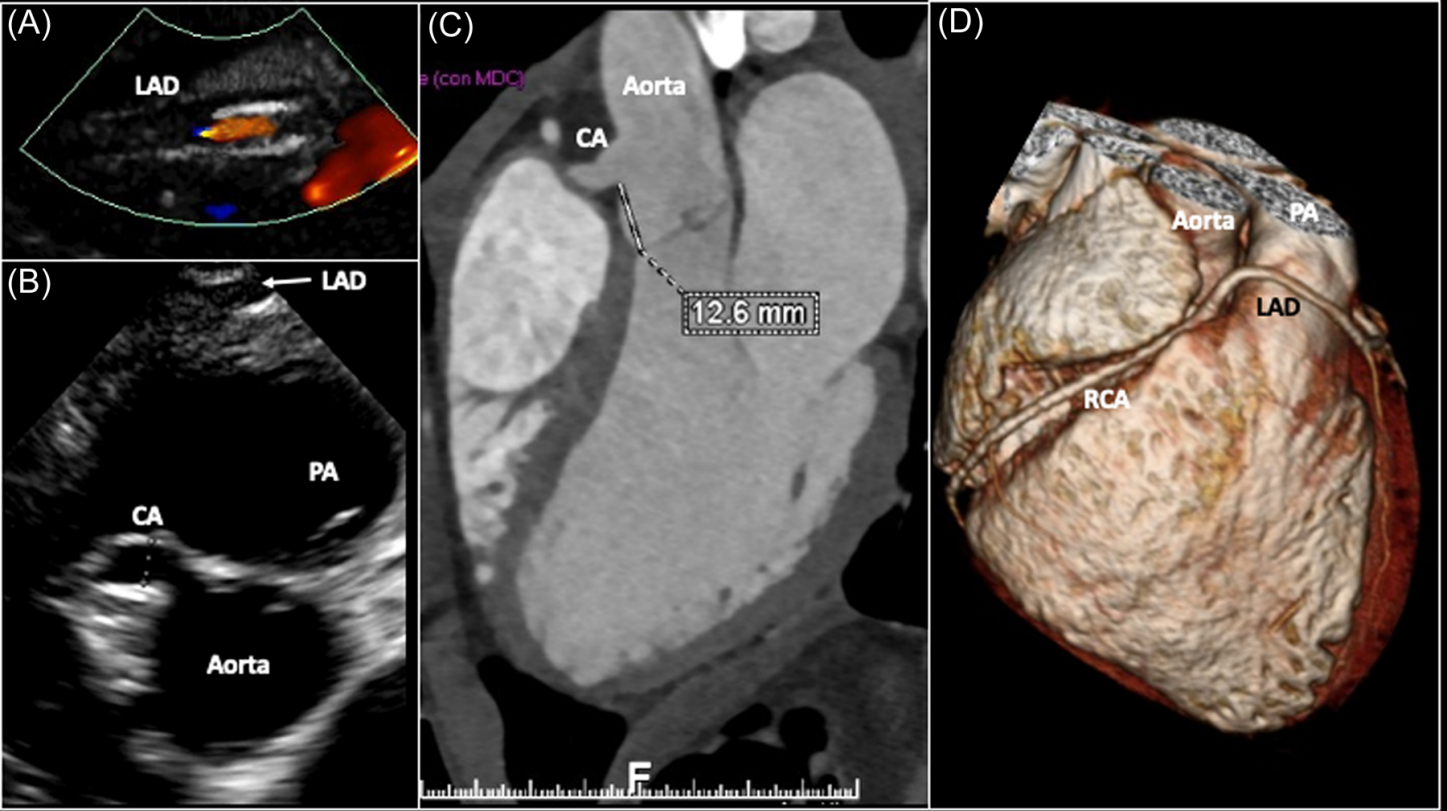
Thirteen year old boy, with a cardiac murmur at the preparticipation screening for competitive swimming. The transthoracic echocardiogram (A and B) showed a dilated coronary artery (transverse diameter 6 mm) originating from the right sinus of Valsalva, with no vessel arising from the left sinus. Anteriorly to the pulmonary artery a vessel can be observed, consisting of the left anterior descending coronary artery. In panel (A), a magnification of this vessel is shown, with color flow Doppler diastolic signal within the vessel. The coronary computed tomography angiogram (C and D) showed a unique coronary artery originating from the right sinus of Valsalva, with the left anterior descending artery originating from the unique coronary artery and running anteriorly at the pulmonary artery. CA, coronary artery; LAD, left anterior descending artery; PA, pulmonary artery; RCA, right coronary artery.

**Figure 3:**
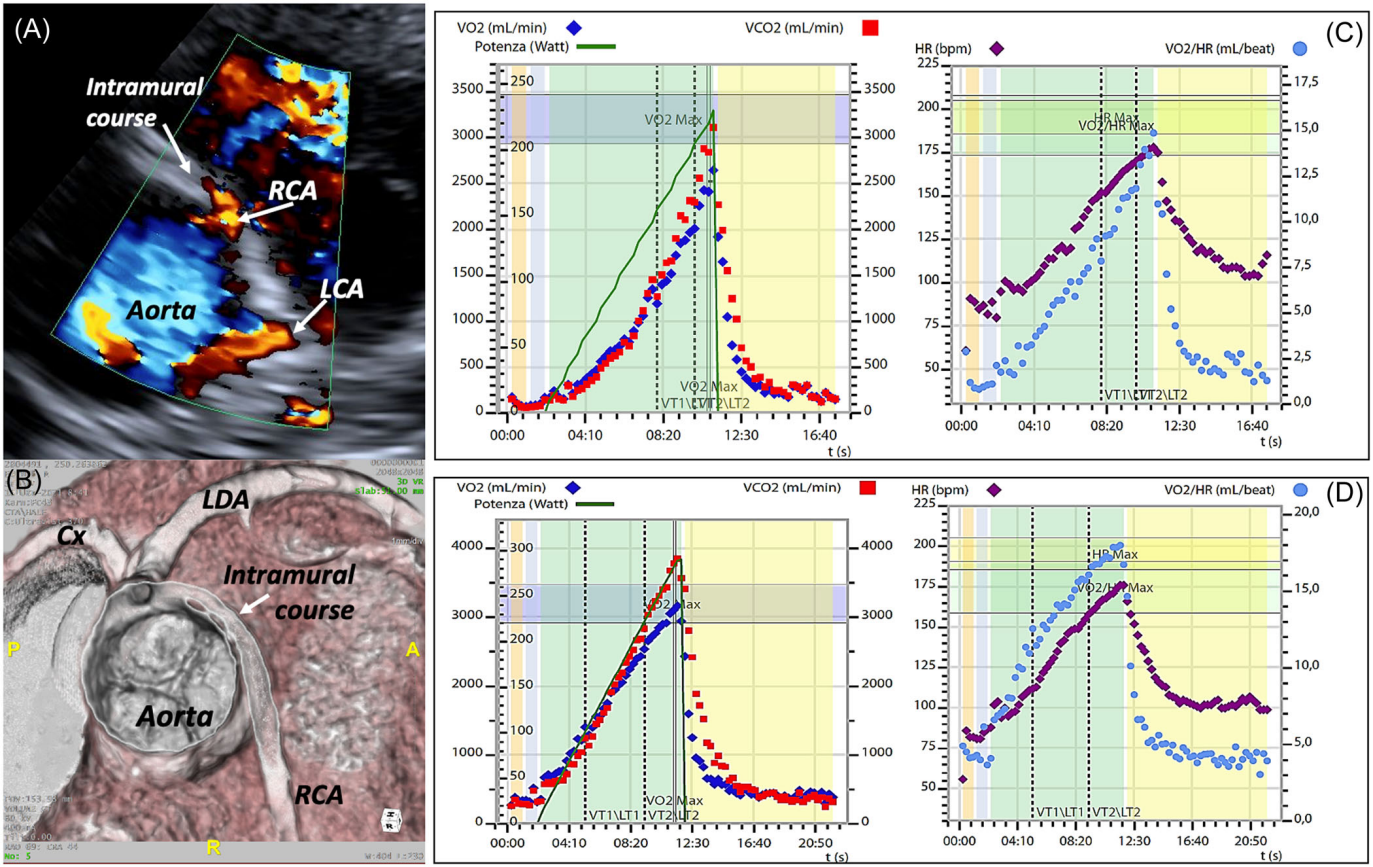
Fourteen years‐old competitive soccer player with presyncope and cold sweating during an official competition. At the bloodwork in the hospital, increased levels of high‐sensitive Troponin were documented. The transthoracic echocardiogram (A) suspected an anomalous origin of the right coronary artery coursing between the aorta and pulmonary artery. The computed tomography angiography (B) confirmed the suspicion, showing an intramural course of the anomalous vessel. Due to the symptoms and imaging risk features, surgery was offered, and the young patient underwent unroofing procedure. Panel (C) shows the cardiopulmonary exercise test (CPET) results before surgery. The oxygen uptake (VO_2_) at peak of the exercise was 37.2 mL/kg/min (73% of the expected) and the oxygen pulse (heart rate/VO_2_) was 14.2 mL/bpm (84% of the expected). Three months after unroofing procedure, following a light aerobic rehabilitation protocol, the CPET results (D) showed an increase of both VO_2_ peak (46.1 mL/kg/min, 91% of the expected) and oxygen pulse (16.9 mL/bpm, 105% of the expected), falling in the normal range for this boy. Cx, circumflex coronary artery; LAD, left anterior descending artery; LCA, left coronary artery; RCA, right coronary artery.

There is not a typical way of presentation for patients with AOCA. In some cases, the first and unique presentation is, unfortunately, aborted, or true SCD. In a significant number of cases, usually asymptomatic, the coronary anomaly is discovered incidentally (Figure 1) on a transthoracic echocardiogram (TTE) or a computed tomography angiogram (CTA) requested for another reason, such as a heart murmur or an abnormal ECG. Finally, there are cases in whom the coronary anomaly is identified by TTE required to investigate unclear symptoms related to physical exercise and performed by cardiologists with specific experience in visualizing the coronary arteries (Figures 2 and 3).

**Figure 4:**
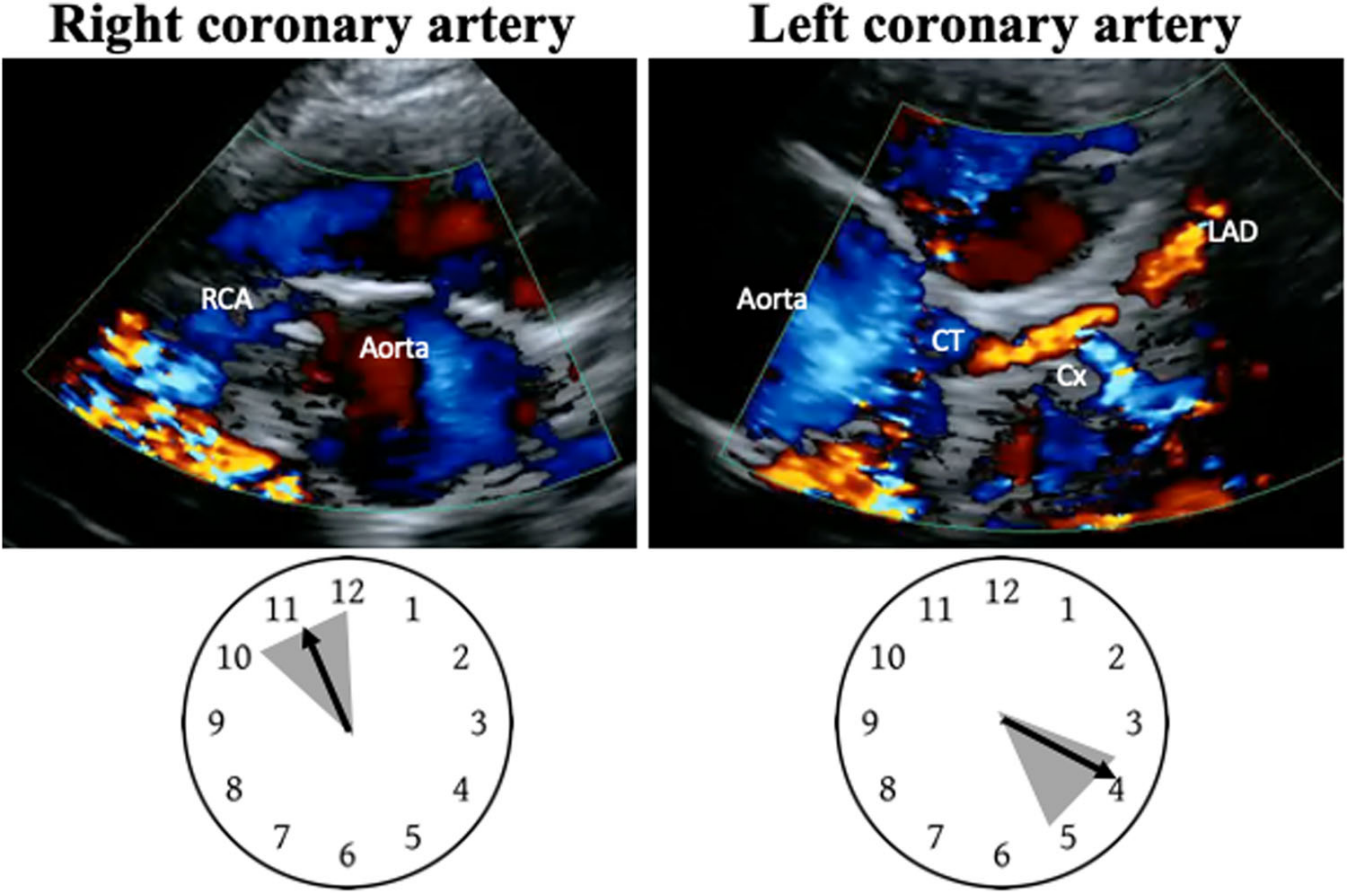
Normal position of the coronary ostia at transthoracic echocardiogram. In a clockwise fashion, the right coronary ostium is normally located at 10−12 o'clock, whilst the left coronary ostium is at 4−5 o'clock. The gray areas in the clock represent the normal position where the coronary ostia can be imaged. The color Doppler flow can be very helpful in detecting the first tracts of the coronary arteries. In the left panel, the right coronary artery appears in blue color and the common trunk is visualized with a first tract in blue and, after a smooth curve, in red color. The circumflex artery is originating from the common trunk and is visualized in light blue color. CT, common trunk; Cx, circumflex coronary artery; LAD, left anterior descending artery; RCA, right coronary artery.

Nowadays, an increasing body of literature confirms that TTE is a useful, reliable method for noninvasive in vivo detection of AOCAs, especially in young athletes, usually with training bradycardia and a good or excellent acoustic windows.^32^, ^33^, ^34^, ^35^, ^36^, ^37^, ^38^, ^39^ However, systematic TTE‐screening of all asymptomatic athletes is neither advisable nor feasible. Instead, it is time to encourage all cardiologists who perform a TTE in an athlete for whatever reason to explore coronary anatomy as much as possible to verify the correct position of the ostia and of the first tracts of the right and left coronary artery in their “appropriate” sinuses. Obviously, the echocardiographic visualization of a correct positioning of the ostia and first tracts of the coronary arteries becomes “strongly recommended” in athletes with abnormal rest and/or stress test ECG, and/or symptomatic for chest pain/discomfort or syncope on effort, or less specific symptoms as palpitations, dizziness, breathlessness. In our experience, a not rare clinical presentation is the history of one or more episodes of cold sweating and/or presyncope during or shortly after effort (Figure 3). In this context, the sports physicians should make a specific request for the search of ostia and first tracts of coronary arteries and TTE should be done by personnel with specific training and expertise in visualizing coronary arteries.^2^, ^36^ Synthetically, the coronary ostia at TTE are better visualized from the parasternal short axis view of the aortic root. If we compare the aortic root perimeter to a clock, the echocardiographer can normally find the right ostium at 10−11 o'clock, with the left ostium at 4−5 o'clock (Figure 4). From this projection, we can also explore the anatomy of the aortic valve, as AOCAs can be associated with a bicuspid aortic valve.^40^ From the same view, slowing tilting the ultrasound probe, the echocardiographer can visualize the first tract of both coronary arteries. At this scope, the Color Doppler imaging may be very helpful in detecting the proximal route of coronary vessels after adequate adjustments of the color flow velocity scale (Figures 1, 2 and 3A). When the visualization of the right coronary first tract is not adequate, a better imaging can be obtained from a lower intercostal space. Conversely, the first tract of the left coronary artery may be better visualized from an upper intercostal space. It is not uncommon, from this position to visualize the origin of the circumflex artery and the color Doppler flow can again be very helpful for this purpose (Figure 4).

In case of TTE suspicion of an AOCA, additional imaging studies, such as cardiac MRI^7^, ^13^, ^41^ and coronary CTA^42^, ^43^ are reasonable to better visualize the coronary artery anatomy and to confirm the diagnosis, with the former being the choice modality in children, to avoid radiation exposure.

This diagnostic algorithm, improved over time, allowed us to publish in 2018 a series of 23 live athletes with AOCA (17 AORCAs)^36^ that have grown to 50 (overall, 35 AAORCAs, 13 AAOLCAs, and 2 single coronary arteries) at the time of writing this paper. To these, we must add another 22 athletes with anomalous origin of the circumflex artery from the right sinus and 55 with myocardial bridging of the left descending artery.

When imaging studies confirm an AOCA, a stress test should be conducted first to evaluate the presence of ischemic ECG changes, that is, mainly ST segment depression, and/or the occurrence of arrhythmias. However, rest ECG is usually normal,^44^ and signs of myocardial ischemia at stress testing are present only in one‐third to half of cases.^1^, ^36^, ^45^, ^46^ Other stress testing modalities include stress echocardiography (both physical or pharmacological), stress nuclear perfusion imaging (NPI), MRI under pharmacological stress (adenosine or dobutamine), but all of them, over time, have frustrated clinicians by producing both low numbers of true‐positive and relatively high numbers of false‐positive results.^47^, ^48^

In particular, stress NPI studies are limited by lower spatial resolution for small defects, attenuation artifacts related to the body wall and diaphragm, relatively high incidence of false positive findings,^49^, ^50^ and potentially harmful effects of ionizing radiations. A promising minimally invasive tool seems to be the stress MRI (aside from its role in confirming the diagnosis of AOCAs as an alternative to coronary CTA).^7^, ^13^, ^41^ MRI may show at rest the presence of myocardial scars as late gadolinium enhancement areas (with ischemic patterns) and, during pharmacological stress, may detect wall motion anomalies in the territory of the anomalous coronary artery. This test has proved to be more accurate than stress echocardiography and has been used both in adults (mainly in coronary artery disease patients) and children.^49^, ^50^, ^51^, ^52^, ^53^, ^54^

However, all the pharmacological stress test modalities increase heart rate (HR) and myocardial contractility through a mechanism other than the physiological one (physical exercise) and do not allow to reach HR as high as those achieved during sports (a young soccer player can easily reach HR above 170−180 beats per min during a match!). This eventually may lead to a possible underestimation of real myocardial ischemia.^55^ In a quite recent paper, Doan et al. used dobutamine stress MRI (when needed with atropine adjunct) in a group of children with AOCA and only 12.1% reached a HR ≥ 85% of their expected maximum, that, in our opinion, should be the minimum target for evaluating an athlete usually engaged in heavy physical exertion. Moreover, the higher the HR, the higher the difficulties in interpreting the imaging results. In the same study, indeed, the authors confirmed that “at HRs >85% of age‐predicted maximum there was a slight shift of the cine sequences' quality from good to adequate; however, both temporal and in‐plane resolution were of diagnostic quality for interpretation.”^56^

A possible alternative to stress testing is stress echocardiography,^57^, ^58^ however with all the limitations reported above for pharmacological stress. Moreover, in case of physical stress during TTE, even if the reached HR is usually higher than the one reached under pharmacological stimulus, it is lower than the HRs reached in standing positions. The adjunct of methodological difficulties in performing a TTE under physical stress (kind of ergometer, body position, and movement, data interpretation, hyperventilation, sweating, pediatric patient's compliance, etc.) makes this diagnostic modality poorly used.

A long‐known functional test that is returning to the attention of the clinical cardiologist is the cardiopulmonary exercise test (CPET). In the past, due to difficulties in the management of the hardware and in the interpretation of the results, it was poorly used in cardiology and relegated in the exercise physiologists' lab. Today, thanks to the technological improvements and more user‐friendly software to analyze functional data, it is starting to be widely used in cardiology, both for adults and children. It has the advantage of a contemporary assessment of stress ECG and respiratory gas analysis, without radiation exposure, providing indexes of cardiac and systolic output (i.e., peak oxygen uptake and oxygen pulse, respectively).^59^, ^60^, ^61^, ^62^ Importantly, it allows reaching the same HR usually reached during physical exercise and in a more physiological way. In our experience, CPET has proved to be of great utility both in the preoperative diagnosis and risk assessment, and in the rehabilitation and return to sport after any surgical correction (Figure 3C,D). In particular, CPET not only provides peak oxygen uptake and peak oxygen pulse values, but the trajectory of these parameters seems to reflect the trend of cardiac and systolic output during the incremental exercise, respectively. When a cycloergometer is used for the CPET, also the oxygen uptake to work rate ratio can be useful in suspecting an inducible myocardial ischemia.^59^, ^60^, ^61^, ^62^

Finally, after an AOCA has been diagnosed in an athlete, basically based on some anatomical features of the anomalous vessel and/or ischemic changes during stress or functional tests, a cardiac catheterization should be performed with some very experienced authors proposing, during this procedure, a routine intravascular ultrasonography evaluation of the anomalous artery for a more precise risk assessment.^23^ In the cath lab (or even during CTA) the evaluation of the fractional flow reserve (FFR) or instantaneous wave‐free ratio (iFR) may be helpful in providing a functional stenosis of the anomalous artery^63^, ^64^, ^65^ and in leading the treatment strategy.

Thus, all these imaging modalities, with crucial role of coronary CTA, are fundamental tools for the risk assessment of athletes with AOCA (Table 1). The presence of ostial stenosis in association with an oblique take‐off from the aorta, ostial ridge, anomalous tract hypoplasia, intussusception, noncompliant pericommissural area, and compression of the anomalous coronary artery intramurally and/or between the great arteries and, in adults, coexistence of atherosclerotic coronary artery disease seem to be key factors, acting alone or in combination, to predispose to myocardial ischemia and/or lethal ventricular arrhythmias.^5^, ^22^, ^36^, ^66^, ^67^, ^68^

**Table 1:** High and low risk features at different diagnostic modalities in anomalous origin of coronary arteries (AOCAs).

AOCA	High risk	Low risk
Clinical features	Chest pain/discomfort	
	Syncope on effort	
	Cold sweating and/or presyncope during or shortly after effort	
	Palpitations, dizziness, breathlessness	
Functional tests (stress test ECG; CPET; NPI; pharmacological stress test)	Signs of myocardial ischemia	
Anatomical findings at imaging (TTE; CTA; cardiac MRI)	AOLCA	Anomalous origin of left common trunk or the left anterior descending coronary artery alone from the opposite sinus running through the conal septum (intraseptal, intraconal, or intramyocardial)
	Intramural course	
	Acute angle take‐off	
	Slit‐like orifice or ostial ridge	
	Hypoplasia of the first tract	
	Oval shape at transverse section of the first tract	
Invasive functional tests (IVUS; coronary angiography FFR/iFR)	FFR ≥0.08; iFR ≥0.89^a^	

*Note*: As anticipated in the introduction of the article, only AOCAs from the opposite sinus of Valsalva with an interarterial course have been considered.

Abbreviations: AOLCA, anomalous origin of the left coronary artery from the opposite sinus of Valsalva; CPET, cardiopulmonary exercise testing; CTA, coronary computed tomography angiography; ECG, electrocardiogram; FFR, fractional flow reserve; iFR, instantaneous wave‐free ratio; IVUS, intra‐vascular ultrasonography; MRI, magnetic resonance imaging; NPI, nuclear perfusion imaging; TTE, transthoracic echocardiography.

^a^ Tajeddini F, Nikmaneshi MR, Firoozabadi B, Pakravan HA, Ahmadi Tafti SH, Afshin H. High precision invasive FFR, low‐cost invasive iFR, or noninvasive CFR?: optimum assessment of coronary artery stenosis based on the patient‐specific computational models. *Int J Numer Method Biomed Eng*. 2020;36(10):e3382.

## 5 MANAGEMENT

Risk‐stratification, treatment strategy (surgery vs. conservative) and decision on sports eligibility in sedentary subjects with AOCA, recreational and competitive athletes, are still challenging for cardiologists and sports physicians.

Our opinion is that there is no standardized, one‐fits‐for‐all protocol, but that we must evaluate all the several aspects mentioned above in each single subject, knowing that it is not always easy to define them with absolute precision, considering the type of AOCA and the presence of symptoms or signs of myocardial ischemia at rest and mostly during functional tests. In addition, we must bear in mind that heavy exercise may trigger totally unexpected SCD,^16^ which is probably the end result of repeated bursts of exercise‐induced acute myocardial ischemia, leading to cumulative myocardial damage, especially in AOLCA though cases of SCD or resuscitated cardiac arrest during exercise are reported also in subjects with AORCA.^1^, ^2^, ^6^, ^69^, ^70^

Following current guidelines, individuals with AOCA and symptoms of ischemic chest pain/discomfort, syncope suspected to be due to life‐threatening ventricular arrhythmias, or a history of aborted SCD, should be restricted from participation in all sports and offered surgery.^17^, ^46^, ^67^, ^71^, ^72^, ^73^, ^74^, ^75^, ^76^, ^77^, ^78^, ^79^, ^80^, ^81^ The same option should be offered to athletes with an AOLCA with an interarterial/intramural course even if asymptomatic and without any sign of myocardial ischemia at stress tests.^17^, ^67^, ^71^, ^72^, ^73^, ^74^, ^79^, ^81^ Surgery for this kind of anomalies should eliminate the intramural course and any associated ostial narrowing by unroofing or coronary translocation with neo‐ostium creation. Repositioning of the pulmonary artery confluence away from the anomalous artery (laterally or anteriorly) may be considered as an adjunctive procedure to the previous, as it is no longer used as the only procedure in the treatment of these anomalies.^78^, ^80^, ^82^, ^83^, ^84^, ^85^ In the uncommon case surgery is not feasible, a catheter‐based intervention may be considered.^22^ If the athlete should refuse surgery, exercise restriction and beta‐blocking therapy seem to be the only alternative.

More problematic is the approach to athletes with AORCA, who are generally considered with lower risk of SCD than individuals with AOLCA. However, the risk exists and can't be properly quantified only based of presence/absence of symptoms or functional testing.^1^, ^2^, ^6^, ^18^, ^69^, ^70^ As stated above, stress NPI or MRI may have a role, but, in the general and our opinion, they suffer from important limitations, especially in pediatric patients.^2^, ^10^, ^36^, ^86^, ^87^, ^88^ New techniques in functional assessment of inducible myocardial ischemia (i.e., FFR during coronary angiography or even CTA and iFR)^63^, ^64^, ^65^ can probably open new perspectives, but conclusive evidence is still lacking.

For these reasons, we believe that risk‐stratification, before and after surgery, should be multiparametric and include all available elements. Anatomical features of the anomalous artery, that is, morphology of the ostium and take‐off angle, length of intra‐arterial/intramural segment, and significant reduction of lumen area (“hypoplasia”), are probably crucial in making one subject “at risk” or not, but we still do not know exactly what is the specific weight of each of them. For this reason, we are adopting a restrictive approach also for competitive athletes with AORCA and an interarterial/intramural course, disqualifying them from sport, although we admit that Italian law, which attributes legal responsibility to sports physicians, partially influences our behavior.

Anyhow, on the basis of our experience and literature data, we think that surgical treatment of AORCA with interarterial/intramural course should ever be considered in case of (a) symptoms during or immediately after exertion; (b) positive stress‐tests for ischemia; (c) presence of an intramural proximal segment and/or critical reduction of lumen area. In other cases, exercise restriction, allowing however moderate recreational physical activity and a medical therapy (beta‐blocking) seem safe enough even if beta‐blocking therapy is not supported by evidence yet.^22^ Nevertheless, in our series with conservative management and beta‐blocking therapy (updated since our publication),^36^ no athlete had recurrence of symptoms or major cardiac events during an average follow‐up of 77 months. However, for these subjects and for those who refuse surgery, more specific counseling concerning the risk of SCD is always mandatory and it should be undertaken with both the athlete and family.

## 6 RETURN TO PLAY AFTER SURGERY

Decision about returning to recreational high‐intensity or competitive sports after surgery requires careful evaluation. Different surgical techniques do not have the same results in removing potential mechanisms that might cause ischemia. Currently, the “unroofing procedure” seems the most used and the more effective in most cases,^70^, ^76^, ^80^, ^89^, ^90^, ^91^ but it can have sequelae and it is not always feasible. Furthermore, in some cases, surgery can only partially resolve the problem.^20^ To our knowledge, just a few cases of exercise SCD after‐surgery have been reported,^20^, ^87^ even if some authors have news of other pediatric deaths postoperatively, that have not been published.^22^ It is interesting to note that, at least in the young, if a patient presents with resuscitated SCD and survives the surgery, he or she may still be at increased risk for a SCD when returning to sport.^87^

Those who have undergone surgery will need close follow‐up in the initial postoperative period: first resting ECG and TTE at 7−10 days from surgery, then at 4−6 weeks with the first functional test at 3 months. If the first controls give favorable results, after the first year from intervention the interval between medical checks can be prolonged.^22^ According to the recent North American guidelines, these patients may return even to competitive sports at least 3 months postoperatively if an exercise stress test reveals no evidence of myocardial ischemia or ventricular arrhythmias.^22^, ^79^, ^92^ Regarding patients who experienced an aborted SCD, it is recommended that they do not return to competitive sport for at least 1 year postoperatively if they are fully asymptomatic and have a negative exercise stress test.^22^

However, all these recommendations come from experts' consensus papers, with a relatively low level of evidence.^22^, ^79^, ^92^ Honestly speaking, we lack long‐term follow‐up of patients and athletes after surgical repair. Short‐ and medium‐term results are encouraging, however there are reports of issues, such as new aortic valve regurgitation, that will need to be followed over time.^22^

In our opinion, we can consider *return to recreational high‐intensity or competitive sport* (Figure 1) after surgery only if: (a) surgery removed all mechanisms potentially responsible for exercise‐induced ischemia; (b) no symptoms/signs of inducible myocardial ischemia and/or significant arrhythmias or sequelae are present at least 6 months after the procedure. For this purpose, we recommend an accurate “post‐surgery protocol” including at least TTE, stress test ECG (we suggest a CPET) and CTA/MRI (preferably both) to evaluate the new anatomy and function of the operated coronary artery.^20^ MRI, in particular, can be a valuable tool in case of the occurrence of ventricular arrhythmias or symptoms (i.e., palpitations, syncope, or presyncope) to rule out a myocardial scar after surgical repair.

After return to competitive sport, we recommend ECG, TTE, and stress test follow‐up every 6 months for the first 3 years from surgery, with Holter monitoring when needed. After this period, the time interval may be prolonged to 1 year if no alterations have been encountered over time.

In all operated cases, however, we recommend that an automated external defibrillator with trained personnel should be immediately available during competition and training, even if this should be the rule in modern sporting setting.^93^, ^94^

Finally, those managed conservatively with exercise restriction will need follow‐up annually.^22^, ^95^, ^96^, ^97^

## CONFLICT OF INTEREST STATEMENT

The authors declare no conflict of interest.

## Data Availability

Data sharing is not applicable to this article as no new data were created or analyzed in this study.
